# Multivariate Multiscale Dispersion Entropy of Biomedical Times Series

**DOI:** 10.3390/e21090913

**Published:** 2019-09-19

**Authors:** Hamed Azami, Alberto Fernández, Javier Escudero

**Affiliations:** 1 School of Engineering, Institute for Digital Communications, University of Edinburgh, King’s Buildings, Edinburgh EH9 3FB, UK; javier.escudero@ed.ac.uk; 2 Department of Neurology and Massachusetts General Hospital, Harvard Medical School, Charlestown, MA 02129, USA; 3 Departamento de Psiquiatría y Psicología Médica, Universidad Complutense de Madrid, 28040 Madrid, Spain; aferlucas@med.ucm.es; 4 Laboratorio de Neurociencia Cognitiva y Computacional, Centro de Tecnología Biomédica, Universidad Politecnica de Madrid and Universidad Complutense de Madrid, 28040 Madrid, Spain

**Keywords:** complexity, multivariate multiscale dispersion entropy, multivariate time series, electroencephalogram, magnetoencephalogram

## Abstract

Due to the non-linearity of numerous physiological recordings, non-linear analysis of multi-channel signals has been extensively used in biomedical engineering and neuroscience. Multivariate multiscale sample entropy (MSE–mvMSE) is a popular non-linear metric to quantify the irregularity of multi-channel time series. However, mvMSE has two main drawbacks: (1) the entropy values obtained by the original algorithm of mvMSE are either undefined or unreliable for short signals (300 sample points); and (2) the computation of mvMSE for signals with a large number of channels requires the storage of a huge number of elements. To deal with these problems and improve the stability of mvMSE, we introduce multivariate multiscale dispersion entropy (MDE–mvMDE), as an extension of our recently developed MDE, to quantify the complexity of multivariate time series. We assess mvMDE, in comparison with the state-of-the-art and most widespread multivariate approaches, namely, mvMSE and multivariate multiscale fuzzy entropy (mvMFE), on multi-channel noise signals, bivariate autoregressive processes, and three biomedical datasets. The results show that mvMDE takes into account dependencies in patterns across both the time and spatial domains. The mvMDE, mvMSE, and mvMFE methods are consistent in that they lead to similar conclusions about the underlying physiological conditions. However, the proposed mvMDE discriminates various physiological states of the biomedical recordings better than mvMSE and mvMFE. In addition, for both the short and long time series, the mvMDE-based results are noticeably more stable than the mvMSE- and mvMFE-based ones. For short multivariate time series, mvMDE, unlike mvMSE, does not result in undefined values. Furthermore, mvMDE is faster than mvMFE and mvMSE and also needs to store a considerably smaller number of elements. Due to its ability to detect different kinds of dynamics of multivariate signals, mvMDE has great potential to analyse various signals.

## 1. Introduction

Multivariate techniques are needed to analyse data consisting of more than one time series [1,2,3]. The majority of physiological and pathophysiological activities, and even many non-physiological signals, include interactions between different kinds of single processes. Thus, we expect that parameters or measures with different origins are considered in a multivariate way [1,4]. Furthermore, recent developments in sensor technology enabling routine recordings of multi-channel signals have led to an increasing popularity of this kind of analysis on physiological data [1,2,3,5,6].

Advances on information theory and non-linear dynamical approaches have recently allowed the study of different kinds of multivariate time series [3,7,8,9]. Due to the intrinsic non-linearity of diverse physiological and non-physiological processes, non-linear analysis of multivariate time series has been broadly used in biomedical signal processing with the aim of studying the relationship between simultaneously recorded signals [3,7,8].

Multivariate multiscale entropy (mvMSE) as a powerful non-linear measure is based on a combination of multivariate sample entropy (SampEn–mvSE) and the coarse-graining process [8]. mvSE characterizes the likelihood that similar multi-channel embedded patterns, which consider both the time and spatial domains, within a time series will remain similar when the pattern length is increased [3]. mvMSE, by taking into account both the spatial and time domains, shows the complexity of multi-channel signals [8]. Complexity reflects the degree of structural richness of time series [8,10] and is different with that of irregularity or uncertainty defined from classical entropy methods such as SampEn [11], permutation entropy (PerEn) [12], and dispersion entropy (DisEn) [13]. That is to say, neither completely regular or certain nor completely irregular (uncorrelated random) time series are truly complex, since none of them is structurally rich at a global level [8,10,14,15,16].

The multivariate multiscale entropy-based analysis is interpreted based on: (1) the multivariate time series **X** is more complex than the multivariate time series **Y**, if for the most temporal scales, the mvSE measures for **X** are larger than those for **Y**; (2) a monotonic fall in the multivariate entropy values along the temporal scale factors shows that the signal only includes useful information at the smallest scale factors; and (3) a multivariate signal illustrating long-range correlations and complex creating dynamics is characterized by either a constant mvSE or this demonstrates a monotonic rise in mvSE with the temporal scale factor [8].

Although the mvMSE is a powerful and widely-used method, when applied to short signals, the results may be undefined or unreliable [17]. To alleviate this shortcoming, multivariate multiscale fuzzy entropy (mvMFE) based on multivariate fuzzy entropy (mvFE) and the coarse-graining process was suggested [18]. To decrease the running time of the mvMFE proposed in [18], we have recently proposed an mvMFE with a new fuzzy membership function [17]. Nevertheless, the mvMFE is still slow for real-time applications and may lead to unreliable results for short signals, as shown later.

To overcome the problem of unreliable values for mvMFE and mvMSE, multivariate multiscale PerEn (mvMPE) was proposed [19]. To have more information regarding the amplitude of multi-channel signals, multivariate weighted multiscale PerEn (mvWMPE) has recently been developed [20]. However, both the mvMPE and mvWMPE do not take into account the cross-statistical properties between multiple input channels and do not follow the concept of complexity for some signals such as white Gaussian noise (WGN) and 1/f noise [8,14,17].

mvMSE and mvMFE have growing appeal and broad use. They have been successfully used in a number of biomedical and mechanical engineering applications, such as, to characterise electroencephalogram (EEG) signals in Alzheimer’s disease (AD) [21,22], to quantitatively distinguish different horizontal oil–water flow patterns [23], to analyze mechanical vibration noise to stimulate the patient’s feet while wearing the shoes [24], to analyze the multivariate cardiovascular time series [25], to characterize focal and non-focal EEG time series [17], to analyze the complexity of interbeat interval and interbreath signals [8], and to analyze the postural fluctuations in fallers and non-fallers older adults [26].

However, mvMSE and mvMFE have the following shortcomings: (1) mvMSE and mvMFE values may be unreliable and unstable for short signals (300 sample points); (2) they are not quick enough for real-time applications; and (3) computation of mvMSE and mvMFE of a signal with a large number of channels needs to have large memory space, as shown later. To address these drawbacks and due to the advantages of multiscale dispersion entropy (DispEn-MDE) over the state-over-the-art multiscale entropy techniques in terms of distinguishing different kinds of dynamics of univariate synthetic and real time series and computation time [27,28,29], we propose four algorithms to extend our recently developed MDE to its multivariate forms, termed multivariate MDE (mvMDE). To evaluate the mvMDE methods, we use both synthetic and real multivariate datasets. Our results indicate that mvMDE is noticeably faster than the existing methods, leads to more stable results, better discriminates different kinds of biomedical time series, does not lead to undefined values for short multivariate time series, and needs to store a considerably smaller number of elements in comparison with mvMSE and mvMFE.

## 2. Multivariate Multiscale Dispersion Entropy (mvMDE)

In this study, we propose and explore three different alternative implementations of mvMDE until we arrive at a fourth and preferred one. All the mvMDE implementations include two main steps: (1) coarse-graining process for multivariate time series; and (2) multivariate DispEn (mvDE), as an extension of our recently developed DisEn [13]. It is worth noting that for all the mvMDE algorithms, the mapping based on the normal cumulative distribution function (NCDF) used in the calculation of mvDE for the first temporal scale factor is maintained fixed across all scales. In fact, in the mvMDE, μ and σ of the NCDF are respectively set at the average and standard deviation (SD) of the original time series and they remain constant for all temporal scale factors. This fact is similar to *r* in the mvMSE and mvMFE, setting at a certain percentage (usually 15%) of the SD of the original signal and remaining constant for all scales [8,17].

### 2.1. Coarse-Graining Process for Multivariate Signals

Assume we have a *p*-channel time series $U={\left\{u_{k,b}\right\}}_{k=1,2,\cdots ,p}^{b=1,2,\cdots ,L}$ of length *L*. In the mvMDE algorithms, for each channel, the original signal is first divided into non-overlapping segments of length τ, named scale factor. Next, for each channel, the average of each segment is calculated to derive the coarse-grained signals as follows [8,17]:

(1) $${x_{k,i}}^{\left(\tau \right)}=\frac{1}{\tau }\sum_{b=(i-1)\tau +1}^{i\tau }u_{k,b},\;1\leq i\leq \left\lfloor \frac{L}{\tau }\right\rfloor =N\;,\;1\leq k\leq p$$

where *N* denotes the length of the coarse-grained signal. The second step of mvMDE is calculating the mvDE of each coarse-grained signal.

### 2.2. Background Information for the mvDE

We build four diverse alternative implementations of mvDE (mvDE_I_ to III and mvDE) until we arrive at a preferred (or optimal) one, i.e., mvDE. However, here, we present all the simpler alternatives (mvDE_I_ to mvDE_III_), since they can still be useful in some settings and allow for clearer comparisons with other current approaches.

#### 2.2.1. mvDE_I_

The mvDE_I_ of the multi-channel coarse-grained time series $X={\left\{x_{k,i}\right\}}_{k=1,2,\cdots ,p}^{i=1,2,\cdots ,N}$, which is based on the mvMPE algorithm [19], is calculated as follows:

(*a*) First, $X={\left\{x_{k,i}\right\}}_{k=1,2,\cdots ,p}^{i=1,2,\cdots ,N}$ are mapped to c classes with integer indices from 1 to c. To this aim, there are a number of linear and nonlinear mapping approaches [30]. The simple linear mapping technique may lead to the problem of assigning the majority of $x_{k,i}$ to limited classes when maximum or minimum values are noticeably larger or smaller than the mean/median value of the image [30]. The weak permanence of DispEn with linear mapping for the characterization of syntactic and real data was illustrated in [13].

A large number of natural processes illustrate a progression from small beginnings that accelerates and approaches a climax over time (e.g., a sigmoid function) [31,32]. When there is not detailed information, a sigmoid function is often used [30,32,33,34]. The choice of sigmoid function in the context of DispEn was discussed in [30]. We here use NCDF as a well-known sigmoid function like in [13]. Note that using NCDF for each channel also deals with the shortcoming of the amplitude values of each of series $x_{k}$ (k=1,2,⋯,p) may be dominated by the components of vectors coming from the time series with the largest amplitudes. The NCDF maps X into $Y={\left\{y_{k,i}\right\}}_{k=1,2,\cdots ,p}^{i=1,2,\cdots ,N}$ from 0 to 1 as follows:

(2) $$y_{k,i}=\frac{1}{\sigma_{k}\sqrt{2\pi }}\int_{-\infty }^{x_{k,i}}e^{\frac{-{\left(t-\mu_{k}\right)}^{2}}{2\sigma_{k}^{2}}}\;dt$$

where $\sigma_{k}$ and $\mu_{k}$ are the SD and mean of time series $x_{k}$, respectively. Then, we use a linear algorithm to assign each $y_{k,i}$ to an integer from 1 to *c*. To do so, for each member of the mapped signal, we use $z_{k,i}^{c}=round\left(c\cdot y_{k,i}+0.5\right)$, where $z_{k,i}^{c}$ denotes the *i*th member of the classified signal in the *k*th channel and rounding involves either increasing or decreasing a number to the next digit. Note that, although this part is linear, the whole mapping approach is non-linear because of the use of NCDF.

(*b*) Time series $z_{k,j}^{m,c}$ are made with embedding dimension *m* and time delay *d* according to $z_{k,j}^{m,c}=\left\{z_{k,j}^{c},z_{k,j+d}^{c},+\cdots +z_{k,j+(m-1)d}^{c}\right\}$, j=1,2,⋯,N−(m−1)d [11,12,13]. Each time series $z_{k,j}^{m,c}$ is mapped to a dispersion pattern $\pi_{v_{0}v_{1}\cdots v_{m-1}}$, where $z_{k,j}^{c}=v_{0}$, $z_{k,j+d}^{c}=v_{1}$,..., $z_{k,j+(m-1)d}^{c}=v_{m-1}$. The number of possible dispersion patterns that can be assigned to each time series $z_{k,j}^{m,c}$ is equal to $c^{m}$, since the signal has *m* members and each member can be one of the integers from 1 to *c* [13].

(*c*) For each channel 1≤k≤p and for each of $c^{m}$ potential dispersion patterns $\pi_{v_{0}\cdots v_{m-1}}$, relative frequency is obtained as follows:

(3) $$\begin{array}{c} p\left(\pi_{v_{0}\cdots v_{m-1}}\right)=\frac{\#\{j\left|j\leq N-\left(m-1\right)d,z_{k,j}^{m,c}\;\mathrm{has}\;\mathrm{type}\;\pi_{v_{0}\cdots v_{m-1}}\right.\}}{(N-(m-1)d)p} \end{array}$$

where # means cardinality. In fact, $p(\pi_{v_{0}\cdots v_{m-1}})$ shows the number of dispersion patterns of $\pi_{v_{0}\cdots v_{m-1}}$ that is assigned to $z_{k,j}^{m,c}$, divided by the total number of embedded signals with embedding dimension *m* multiplied by the number of channels.

(*d*) Finally, based on the Shannon’s definition of entropy, the mvDE_I_ is calculated as follows:

(4) $$\begin{array}{c} mvDE_{I}\left(X,m,c,d\right)=-\sum_{\pi =1}^{c^{m}}p(\pi_{v_{0}\cdots v_{m-1}})\cdot \ln\left(p(\pi_{v_{0}\cdots v_{m-1}})\right) \end{array}$$

In case all possible dispersion patterns have equal probability value, the highest value of mvDE_I_ is obtained, which has a value of $ln(c^{m})$. In contrast, if there is only one $p(\pi_{v_{0}\cdots v_{m-1}})$ different from zero, which demonstrates a completely regular/certain signal, the smallest value of mvDE_I_ is obtained. In the algorithm of mvDE_I_, we compare Np dispersion patterns of a *p*-channel signal with $c^{m}$ potential patterns. Thus, at least $c^{m}+Np$ elements are stored.

To work with reliable statistics to calculate MDE, it was recommended $c^{m}<\left\lfloor \frac{L}{\tau_{max}}\right\rfloor$ [27]. Since mvDE_I_ counts the dispersion patterns for every channel of a multivariate time series, it is suggested $c^{m}<\left\lfloor \frac{pL}{\tau_{max}}\right\rfloor$. mvDE_I_ extracts the dispersion patterns from each of channels regardless of their cross-channel information. Thus, mvDE_I_ works appropriately when the components of a multivariate signal are statistically independent. However, the mvDE_I_ algorithm, like mvPE [19], does not consider the spatial domain of time series. To overcome this problem, we propose mvDE_II_ based on the Taken’s theorem [17,35].

#### 2.2.2. mvDE_II_

The algorithm of mvDE_II_ is as follows: 

(*a*) First, like mvDE_I_, $X={\left\{x_{k,i}\right\}}_{k=1,2,\cdots ,p}^{i=1,2,\cdots ,N}$ are mapped to $Z={\left\{z_{k,i}\right\}}_{k=1,2,\cdots ,p}^{i=1,2,\cdots ,N}$ based on the NCDF.

(*b*) To take into account both the spatial and time domains, multi-channel embedded vectors are generated according to the multivariate embedding theory [35]. The multivariate embedded reconstruction of **Z** is defined as:

(5) $$\begin{array}{c} Z_{m}\left(j\right)=[z_{1,j},z_{1,j+d_{1}},\cdots ,z_{1,j+\left(m_{1}-1\right)d_{1}},\\ z_{2,j},z_{2,j+d_{2}},\cdots ,z_{2,j+\left(m_{2}-1\right)d_{2}},\cdots ,\\ z_{p,j},z_{p,j+d_{p}},\cdots ,z_{p,j+\left(m_{p}-1\right)d_{p}}] \end{array}$$

where $m=[m_{1},m_{2},\cdots ,m_{p}]$ and $d=[d_{1},d_{2},\cdots ,d_{p}]$ denote the embedding dimension and the time lag vectors, respectively. Note that the length of $Z_{m}\left(j\right)$ is $\sum_{k=1}^{p}m_{k}$. For simplicity, we assume $d_{k}=d$ and $m_{k}=m$, that is, all the embedding dimension values and all the delay values are equal.

(*c*) Each series $Z_{m}\left(j\right)$ is mapped to a dispersion pattern $\pi_{v_{0}v_{1}\cdots v_{mp-1}}$, where $z_{1,j}^{c}=v_{0}$, $z_{1,j+d}^{c}=v_{1}$,..., $z_{p,j+(m-1)d}=v_{mp-1}$. The number of possible dispersion patterns that can be assigned to each time series $Z_{m}\left(j\right)$ is equal to $c^{mp}$, since the signal has mp members and each member can be one of the integers from 1 to *c*.

(*d*) For each of $c^{mp}$ potential dispersion patterns $\pi_{v_{0}\cdots v_{mp-1}}$, relative frequency is obtained based on the DisEn algorithm [13] as follows:

(6) $$\begin{array}{c} p\left(\pi_{v_{0}\cdots v_{mp-1}}\right)=\frac{\#\{j\left|j\leq N-\left(m-1\right)d,Z_{m}\left(j\right)\;\mathrm{has}\;\mathrm{type}\;\pi_{v_{0}\cdots v_{mp-1}}\right.\}}{N-(m-1)d} \end{array}$$

(*e*) Finally, based on the Shannon’s definition of entropy, the mvDE_II_ is calculated as follows:

(7) $$\begin{array}{c} mvDE_{II}\left(X,m,c,d\right)=-\sum_{\pi =1}^{c^{mp}}p(\pi_{v_{0}\cdots v_{mp-1}})\cdot \ln\left(p(\pi_{v_{0}\cdots v_{mp-1}})\right) \end{array}$$

In the algorithm of mvDE_II_, at least $c^{mp}+Np$ elements are stored. Thus, when *p* is large, the algorithm needs huge space of memory to store elements. To work with reliable statistics to calculate mvMDE_II_, it is recommended $c^{mp}<\left\lfloor \frac{L}{\tau_{max}}\right\rfloor$. Thus, although mvDE_II_ deals with both the spatial and time domains, the length of a signal and its number of channels should be very large and small, respectively, to reliably calculate mvDE_II_ values. To alleviate the problem, we propose mvDE_III_.

#### 2.2.3. mvDE_III_

The algorithm of mvDE_III_ is as follows: 

(*a*) First, like the mvDE_I_ and mvDE_II_ approaches, $X={\left\{x_{k,i}\right\}}_{k=1,2,\cdots ,p}^{i=1,2,\cdots ,N}$ are mapped to $Z={\left\{z_{k,i}\right\}}_{k=1,2,\cdots ,p}^{i=1,2,\cdots ,N}$.

(*b*) Multivariate embedded vectors $Z_{k,m}\left(j\right)$ with length m+p−1 are generated according to the Taken’s embedding theorem [35] with *p* embedding dimension vectors $m_{k}=\left[1,1,\cdots ,m_{k},\cdots ,1,1\right]$ (k=1,⋯,p), where $m_{k}$ denotes the $k^{th}$ element of **m**. For simplicity, we assume $m_{k}=m$ and $d_{k}=d$.

(*c*) Each series $Z_{k,m}\left(j\right)$ is mapped to a dispersion pattern $\pi_{v_{0}v_{1}\cdots v_{m+p-2}}$. The number of possible dispersion patterns that can be assigned to each time series $Z_{k,m}\left(j\right)$ is equal to $c^{m+p-1}$, since the signal has m+p−1 members and each member can be one of the integers from 1 to *c* [13]. Since we count the number of patterns for each of *p* different $m_{k}$ leading to a considerable increase in the number of dispersion patterns, compared with mvDE_II_, we have more reliable results for a signal with a small number of samplthan those fore points, as shown later.

(*d*) For each channel 1≤k≤p and for each of $c^{m+p-1}$ potential dispersion patterns $\pi_{v_{0}\cdots v_{m+p-2}}$, relative frequency is obtained as follows:

(8) $$\begin{array}{c} p\left(\pi_{v_{0}\cdots v_{m+p-2}}\right)=\frac{\#\{j\left|j\leq N-\left(m-1\right)d,Z_{k,m}\left(j\right)\;\mathrm{has}\;\mathrm{type}\;\pi_{v_{0}\cdots v_{m+p-2}}\right.\}}{(N-(m-1)d)p} \end{array}$$

(*e*) Finally, based on the Shannon’s definition of entropy, the mvDE_III_ is calculated as follows:

(9) $$\begin{array}{c} mvDE_{III}\left(X,m,c,d\right)=-\sum_{\pi =1}^{c^{m+p-1}}p(\pi_{v_{0}\cdots v_{m+p-2}})\cdot \ln\left(p(\pi_{v_{0}\cdots v_{m+p-2}})\right) \end{array}$$

mvDE_III_ assumes embedding dimension 1 for all signals except one, which might limit the potential to explore the dynamics. Moreover, in the algorithm of mvDE_III_, at least $c^{m+p-1}+Np$ elements are stored. Although this number is noticeably smaller than that for mvDE_II_, the algorithm still needs to have large memory space for a signal with a large number of channels. To work with reliable statistics to calculate mvMDE_III_, it is recommended $c^{m+p-1}<\left\lfloor \frac{pL}{\tau_{max}}\right\rfloor$. Therefore, albeit mvDE_III_ takes into account both the spatial and time domains and needs to smaller number of sample points in comparison with mvDE_II_, there is a need to have a large enough number of samples and small number of channels. To alleviate these deficiencies, we propose mvDE.

### 2.3. Multivariate Dispersion Entropy (mvDE)

The mvDE algorithm is as follows: 

(*a*) First, like mvDE_I_ to III, the multivariate signal $X={\left\{x_{k,i}\right\}}_{k=1,2,\cdots ,p}^{i=1,2,\cdots ,N}$ is mapped to c classes with integer indices from 1 to c.

(*b*) Like mvDE_II_, to consider both the spatial and time domains, multivariate embedded vectors $Z_{m}\left(j\right),1\leq j\leq N-\left(m-1\right)d$ are created based on the Taken’s embedding theorem [35]. For simplicity, we assume $d_{k}=d$ and $m_{k}=m$.

(*c*) For every $Z_{m}\left(j\right)$, all combinations of the $\sum_{k=1}^{p}m_{k}$ elements in $Z_{m}\left(j\right)$ taken *m* at a time, termed $\phi_{q}\left(j\right)$ (q=1,…mpm), are created. The number of the combinations is equal to mpm. Therefore, for all channels, we have (N−(m−1)d)mpm dispersion patterns.

(*d*) For each 1≤q≤mpm and for each of $c^{m}$ potential dispersion patterns $\pi_{v_{0}\cdots v_{m-1}}$, relative frequency is obtained as follows:

(10) p(πv0⋯vm−1)=#{jj≤N−(m−1)d,ϕq(j)hastypeπv0⋯vm−1}(N−(m−1)d)mpm

(*e*) Finally, based on the Shannon’s definition of entropy, the mvDE is calculated as follows:

(11) $$\begin{array}{c} mvDE\left(X,m,c,d\right)=-\sum_{\pi =1}^{c^{m}}p(\pi_{v_{0}\cdots v_{m-1}})\cdot \ln\left(p(\pi_{v_{0}\cdots v_{m-1}})\right) \end{array}$$

In fact, mvDE explores all combinations of patterns of length *m* within an *mp*-dimensional embedding vector. In the mvDE algorithm, at least $c^{m}+Np$ elements are stored. This number is noticeably smaller than those for mvDE_II_ to III, leading to more stable results for signals with a short length and a large number of samples. As the number of patterns obtained by the mvMDE method is (N−(m−1)d)mpm, it is suggested cm<Lmpmτmax to work with reliable statistics. It is worth mentioning that if the order of channels in a multi-channel time series changes, although the assignment to each dispersion pattern obtained by the mvMDE-based methods may change, the entropy value will stay the same.

### 2.4. Parameters of the mvMDE, mvMSE, and mvMFE Methods

In addition to the maximum scale factor $\tau_{max}$ described before, there are three other parameters for the mvMDE methods, including the embedding dimension vector **m**, number of classes *c*, and time delay vector d. Although some information with regard to the frequency of signals may be ignored for $d_{k}>1$, it is better to set $d_{k}>1$ for oversampled time series. However, like previous studies about multivariate entropy methods [2,8], we set $d_{k}=1$ for simplicity. Nevertheless, when the sampling frequency is considerably larger than the highest frequency component of a time series, the first minimum or zero crossing of the autocorrelation function or mutual information can be utilized for the selection of an appropriate time delay [36]. We need 1<c to keep away the trivial case of having only one dispersion pattern. For simplicity, we use c=5 and $m_{k}=2$ for all signals used in this study, although the range 2<c<9 leads to similar findings. For more information about *c*, $m_{k}$, and $d_{k}$, please refer to [13,30].

In this study, $d_{k}$, $m_{k}$, and *r* for the mvMSE and mvMFE were respectively set as 1, 2, and 0.15 of the SD of the original time series following recommendations in [8,17]. The maximum scale factor for mvMSE and mvMFE also follows [8,17]. In the algorithm of mvSE and mvFE, at least Np2+Np(pm+1) elements are stored (the mvSE code available at http://www.commsp.ee.ic.ac.uk/~mandic/research/Complexity_Stuff.htm). Matlab codes of mvMFE and mvMSE are available at http://dx.doi.org/10.7488/ds/1432. Overall, the characteristics and limitations of the mvSE, mvFE, and mvDE algorithms for a *p*-channel signal with length *N* are summarized in Table 1.

## 3. Evaluation Signals

In this section, the descriptions of correlated and uncorrelated noise signals, bivariate autoregressive (BAR) process, and real time series used in this study are given.

### 3.1. Synthetic Signals

The irregularity of multivariate 1/f noise is lower than multivariate WGN, whereas the complexity of the former is higher than the latter [8,14,17]. Thus, 1/f noise and WGN signals have been commonly used to assess the multivariate multiscale entropy techniques [8,17,37]. For more information about the algorithms used for multivariate 1/f noise and WGN, please refer to [8,17].

To understand the behaviour of the mvMDE methods on uncorrelated WGN and 1/f noise, we first generated a trivariate time series, where originally all three data channels were realization of mutually independent 1/f noise. Then, we gradually decreased the number of data channels representing 1/f noise (from 3 to 0) and at the same time, increased the number of variates representing independent WGN (from 0 to 3) [37]. The number of channels was always three.

To create correlated bivariate noise time series, we first generated a bivariate uncorrelated random time series **H**. Afterwards, **H** was multiplied with the standard deviation (hereafter, sigma) and then, the value of the mean (hereafter, mu) was added. Next, **H** was multiplied by the upper triangular matrix **L** obtained from the Cholesky decomposition of a defined correlation matrix **R** (which is positive and symmetric) to set the correlation. Here, we set $R=\left[\begin{array}{cc} 1 & 0.95\\ 0.95 & 1 \end{array}\right]$ according to [8,17]. An in-depth study on the effect of correlated and uncorrelated 1/f noise and WGN on multiscale entropy approaches can be found in [8,10].

Based on the fact that the larger the order of an autoregressive process, the more complex the AR process [8], we evaluate the mvMDE, mvMSE, and mvMFE methods on a BAR(α) process with the maximum lag α describing the evolution of a set of two variables as a linear function of their past values according to:

(12) $$y_{n}=e_{n}+\sum_{\gamma =1}^{\alpha }y_{n-\gamma }A_{\gamma }$$

where $y_{n}=\left\{y_{n}\left(1\right),y_{n}\left(2\right)\right\}$ is the *n*^th^ sample of a bidimensional time series, $A_{\gamma }$ denotes the 2×2 matrix of parameters corresponding to lag order γ, and $e_{n}$ is the 2×1 vector of error terms assumed to be WGN [38].

### 3.2. Real Biomedical Datasets

(*1) Dataset of Stride Interval Fluctuations*: To investigate the ability of the proposed mvMDE methods to reveal the long-range correlations and dynamics of multivariate signals, the stride interval recordings are used [2,39]. The time series were recorded from ten young, healthy men. Mean age was 21.7 years, changing from 18 to 29 years. Height and weight were 1.77 ± 0.08 meters (mean ± SD) and 71.8 ± 10.7 kg (mean ± SD), respectively. All ten participants provided informed written consent walking for 1 hour at slow, 1 hour at normal, and 1 hour at fast paces and also walking a metronome set to each subject’s mean stride interval. Three walking paces were considered as different variables from the same system. In this way, we expect to be able to discriminate between the metronomically-paced and self-spaced walking. For further information, please refer to [39].

(*2) Dataset of Focal and Non-focal Brain Activity*: The ability of the mvMDE methods, in comparison with mvMFE and mvMSE, to differentiate focal from non-focal recordings is evaluated using a publicly-available EEG dataset [40]. The dataset includes 5 patients and, for each patient, there are 750 focal and 750 non-focal bivariate signals. The length of each recording was 20 s with sampling frequency of 512 Hz (10,240 sample points). Further information can be found in [40]. Before computing the aforementioned methods, all recordings were digitally filtered employing an FIR band-pass filter with cut-off frequencies at 0.5 Hz and 40 Hz.

(*3) Surface MEG Recordings in Alzheimer’s Disease*: We analysed resting state MEG time series recorded with a 148-channel whole-head magnetometer. All 62 participants agreed for the research, which was approved by the local ethics committee. To screen the cognitive status, a mini-mental state examination (MMSE) was done. There were 36 AD patients (age = 74.06±6.95 years, all data given as mean ± SD, and MMSE score = 18.06±3.36) and 26 controls (age = 71.77±6.38 years, and MMSE score = 28.88±1.18). The difference in age between two groups was not significant (*p*-value = 0.1911, Student’s *t*-test) [41]. The distribution of MEG sensors is shown in Figure 2 in [41]. For each participant, five minutes of MEG resting state activity were recorded at a sampling frequency of 169.5 Hz. The signals were divided into 10 s segments (1695 samples) and visually inspected using an automated thresholding procedure to discard epochs noticeably contaminated with artifacts. All recordings were digitally band-pass filtered with a Hamming window FIR filter of order 200 and cut-off frequencies at 1.5 and 40 Hz. For more information, please see [41].

## 4. Results and Discussions

### 4.1. Synthetic Signals

#### 4.1.1. Uncorrelated White Gaussian and 1/f Noises

**Figure 1:**
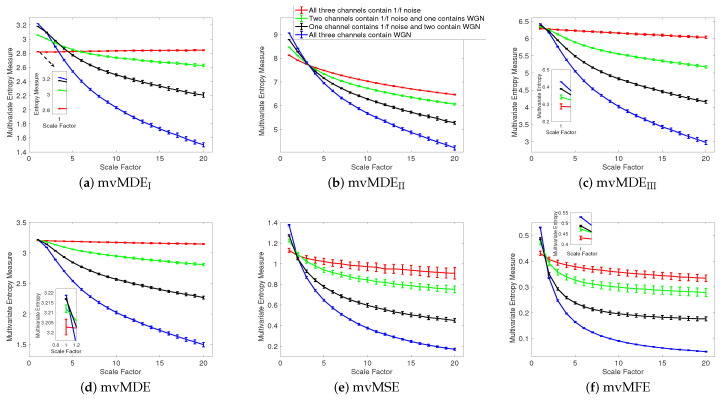
Mean value and SD of the results using (**a**) mvMDE_I_, (**b**) mvMDE_II_, (**c**) mvMDE_III_, (**d**) mvMDE, (**e**) mvMSE, and (**f**) mvMFE computed from 40 different uncorrelated trivariate WGN and 1/f noise time series with length 15,000 sample points.

We first apply the proposed and existing methods to 40 independent realizations of uncorrelated trivariate WGN and 1/f noise, described in Section 3. The number of sample points for each of the 1/f noise and WGN signals were 15,000. mvMSE and mvMFE are based on conditional entropy [2,8,17]. On the other hand, mvMDE is based on the Shannon’s entropy definition applied to dispersion patterns. This means that the methods work on slightly different principles. However, the comparison of mvMDE with mvMSE and mvMFE is meaningful because the latter two are the most common multivariate entropy algorithms and MDE has been shown to have similar behaviour to MSE when analysing real and synthetic signals [27]. Thus, we compare the mvMDE methods with mvMSE and mvMFE. The average and SD of the results for mvMDE_I_, mvMDE_II_, mvMDE_III_, mvMDE, mvMSE, and mvMFE are depicted in Figure 1a–f, respectively. Using all the existing and proposed methods, the entropy values of trivariate WGN signals are higher than those of the other trivariate time series at low scale factors. However, the entropy values for the coarse-grained trivariate 1/f noise signals stay almost constant or decrease slowly along the temporal scale factor, while the entropy values for the coarse-grained WGN signal monotonically decreases with the increase of scale factors. When the length of WGN signals, obtained by the coarse-graining process, decreases (i.e., the scale factor increases), the mean value of inside each signal converges to a constant value and the SD becomes smaller. Therefore, no new structures are revealed at higher temporal scales. This demonstrates a multivariate WGN time series has information only in small temporal scale factors. In contrast, for trivariate 1/f noise signals, the mean value of the fluctuations inside each signal does not converge to a constant value.

For all the methods, the higher the number of variates representing 1/f noise, the higher complexity the trivariate signal, in agreement with the fact that multivariate 1/f noise is structurally more complex than multivariate WGN [8,14,17]. Here, for multivariate 1/f noise and WGN, $\tau_{max}$ was 20 for mvMDE, according to Section 2.

To compare the results obtained by the mvMDE, mvMSE, and mvMFE methods, we used the coefficient of variation (CV). CV, as a measure of relative variability, is defined as the SD divided by the mean of a time series. We use such a metric as the SDs of time series may increase or decrease proportionally to the mean. We investigate the results obtained by uncorrelated noise signals at scale factor 10, as a trade-off between short and long scale factors. As can be seen in Table 2, the smallest CV values for uncorrelated trivariate 1/f noise, an uncorrelated combination of bivariate 1/f noise and univariate WGN, an uncorrelated combination of bivariate WGN and univariate 1/f noise, and trivariate WGN are achieved by mvMDE, mvMDE_II_, mvMDE_II_, and mvMDE_I_, respectively. Overall, the smallest CV values for trivariate 1/f noise and WGN profiles are reached by the mvMDE methods, showing the superiority of the mvMDE methods over mvMSE and mvMFE in terms of stability of results.

**Figure 2:**
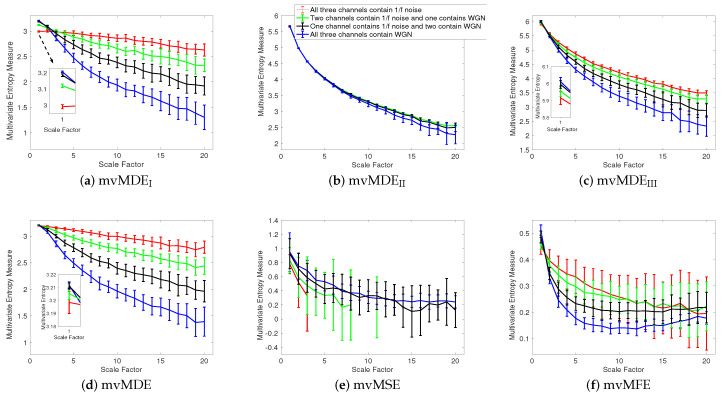
Mean value and SD of the results obtained by (**a**) mvMDE_I_, (**b**) mvMDE_II_, (**c**) mvMDE_III_, (**d**) mvMDE, (**e**) mvMSE, and (**f**) mvMFE computed from 40 different uncorrelated trivariate WGN and 1/f noise time series with length 300 sample points.

To assess the ability of the mvMDE methods to characterize short signals in comparison with mvMFE and mvMSE, we use trivariate 1/f noise and WGN with length of 300 sample points. The results for the mvMDE, mvMSE, and mvMFE approaches at temporal scales 1 to 20 are depicted in Figure 2a–f, respectively. The results show that only mvMDE_I_ is able to distinguish these four different kinds of noise signals at scale factor 1. For the higher temporal scale factors, mvMDE_I_ and mvMDE distinguish these time series, showing a limitation of mvMDE for the discrimination of white from 1/f noise at lower scale factors and also the importance of considering higher temporal scales for the mvMDE technique. As can be seen in Figure 2a,d, the mvMDE_I_ and mvMDE methods better discriminate different dynamics of the noise signals. However, the mvMSE values are undefined at higher scale factors. It is worth mentioning that we compared mvMDE with the original algorithms of mvMSE and mvMFE. However, more recent studies on entropy estimation of short physiological signals provided methods to deal with this issue [17,42].

Although the mvMFE- and mvMDE_II_-based values are defined at all scale factors, they cannot distinguish the dynamics of different noise signals. The profiles obtained by mvMDE_III_ are more distinguishable than mvMDE_II_, as mentioned that mvMDE_III_ needs a smaller number of sample points. Nevertheless, the profiles obtained by mvMDE_III_ have overlaps at several scale factors. Overall, the results show the superiority of mvMDE_I_ and mvMDE over mvMDE_II_, mvMDE_III_, mvMSE, and mvMFE for short uncorrelated signals.

#### 4.1.2. Computational Time

To evaluate the computational time of mvMSE, mvMFE, mvMDE_I_ to III, and mvMDE, we use uncorrelated multivariate WGN time series with different lengths, changing from 100 to 10,000 sample points, and different number of channels, changing from 2 to 8. The results are depicted in Table 3. The simulations have been carried out using a PC with Intel (R) Core (TM) i7-7820X CPU, 3.6 GHz and 16-GB RAM by MATLAB R2018b. The results show that the computation times for mvMSE and mvMFE are close. The slowest algorithm is mvMDE_II_, while the fastest ones are mvMDE_I_ and mvMDE, in that order. For an 8-channel signal with 10,000 samples, using mvMDE_II_, the array exceeded the memory available. Overall, in terms of computation time and memory space, mvMDE outperforms the other methods that take into account both the time and spatial domains. We used the mvMSE code provoided in [8] and the mvMDE, mvMSE, and mvMFE Matlab codes have not been optimized.

#### 4.1.3. Correlated white Gaussian and 1/f Noises

**Figure 3:**
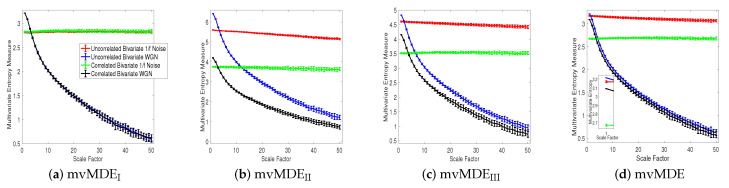
Mean value and SD of the results obtained by (**a**) mvMDE_I_, (**b**) mvMDE_II_, (**c**) mvMDE_III_, and (**d**) mvMDE computed from 40 different correlated and uncorrelated bivariate WGN and 1/f noise time series with length 20,000 sample points.

Univariate multiscale entropy approaches only consider every data channel separately and fail to take into account the cross-channel information of multivariate time series [8]. Uncorrelated multi-channel WGN has less structural complexity and more irregularity compared with multi-channel 1/f noise. To assess the ability of the existing and proposed multivariate entropy methods to reveal the dynamics across the channels, we created 40 independent realizations of different combinations of bivariate 1/f noise and WGN time series with length 20,000 (according to [8,17]), making the channels correlated. Figure 3a–d respectively show the results obtained using the mvMDE_I_, mvMDE_II_, mvMDE_III_, and mvMDE to model both the within- and cross-channel properties in multivariate signals.

mvMDE_I_ cannot discriminate the correlated from uncorrelated WGN or 1/f noise. This fact is revealed in Figure 3a. Therefore, mvMDE_I_ should only be used when the components of a multi-channel time series are statistically independent. Multivariate multiscale entropy-based methods at scale factor 1 show the irregularity of multi-channel signals [8]. The mvMDE_II_, mvMDE_III_, and mvMDE values at scale 1 show that the uncorrelated WGN is the most irregular and unpredictable time series in agreement with [10], while the most irregular signals using mvMFE and mvMSE are the correlated WGN [8,17], in contrast with the fact that correlated multi-channel WGN signals are more predictable and regular than uncorrelated WGN ones [10,27]. Although mvMDE was able to distinguish all four different kinds of noises at the small scale factors, there are some overlaps between the results for the correlated and uncorrelated bivariate WGN time series at the high scale factors showing the importance both low and high temporal scale factors in mvMDE.

The correlated bivariate 1/f noise is the most complex signal using the mvMDE_II_, mvMDE_III_, and mvMDE. The second most complex signal is the uncorrelated bivariate 1/f noise, as can be seen in Figure 3. The decreases of the uncorrelated bivariate WGN profiles using mvMDE_II_, mvMDE_III_, and mvMDE are the largest, evidencing the fact that the uncorrelated WGN is the least complex time series. These facts are also in agreement with the previous studies [8,14,17]. Therefore, as desired, the mvMDE_II_, mvMDE_III_, and mvMDE deal with both the cross- and within-channel correlations.

#### 4.1.4. Bivariate AR Processes

**Figure 4:**
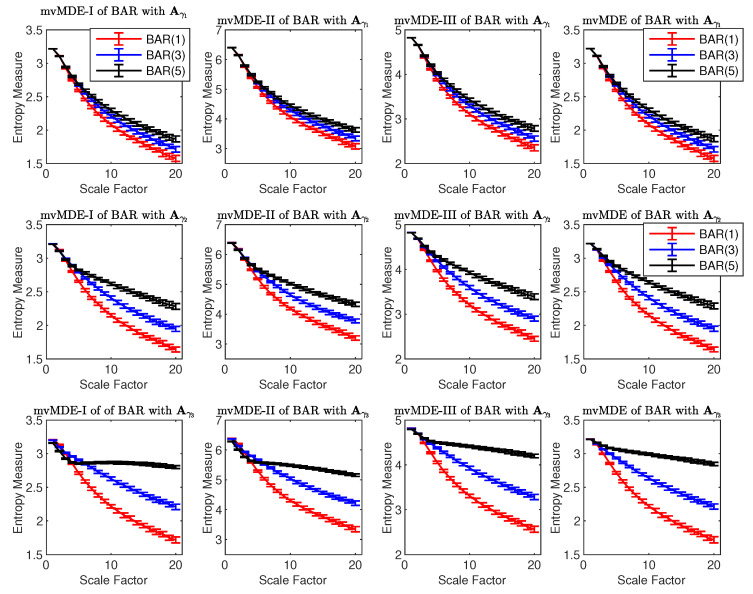
Mean and SD values of the results using mvMDE_I_, mvMDE_II_, mvMDE_III_, and mvMDE computed from 40 different BAR(1), BAR(3), and BAR(5) time series with $A_{\gamma_{1}}$ (first row), $A_{\gamma_{2}}$ (second row), and $A_{\gamma_{3}}$ (third row).

The ability of the mvMDE methods to characterize multivariate AR processes is further evaluated using combinations of BAR(1), BAR(3), and BAR(5) with $A_{\gamma_{1}}=\left[\begin{array}{cc} 0.05 & 0.05\\ 0.05 & 0.05 \end{array}\right]$, $A_{\gamma_{2}}=\left[\begin{array}{cc} 0.10 & 0.10\\ 0.10 & 0.10 \end{array}\right]$, and $A_{\gamma_{3}}=\left[\begin{array}{cc} 0.15 & 0.15\\ 0.15 & 0.15 \end{array}\right]$. The results obtained by the mvMDE_I_, mvMDE_II_, mvMDE_III_, and mvMDE methods are shown in Figure 4. As expected, when the lag order increases, the complexity of the corresponding time series using the mvMDE approaches increases, in agreement with the fact that a larger lag order denotes a more complex time series [8]. As the elements of $A_{\gamma_{1}}$ are smaller than those of $A_{\gamma_{2}}$ and $A_{\gamma_{3}}$, the behaviour of the profiles obtained by the mvMDE methods are more similar to the results for WGN (see Figure 1). In fact, the smaller the elements of $A_{\gamma }$, the less complex the BAR, leading to lower entropy values at higher scale factors.

**Figure 5:**
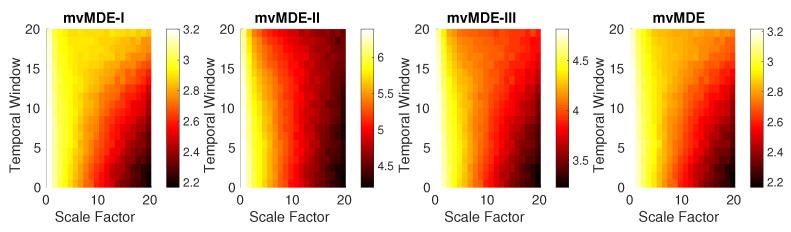
Results obtained by the mvMDE methods using a bivariate temporal window with length 2000 sample points moving along the BAR(3) signal, which the elements of anti-diagonal of the matrix **A** linearly increase from 0 to 0.17, leading to more complex series.

In order to investigate the dependence of the mvMDE methods on the sensitivity to changes in the signals, we generated BAR(3) with length of 10,000 sample points and sampling frequency of 150 Hz that $A_{\gamma }$ linearly changes from $\left[\begin{array}{cc} 0.17 & 0\\ 0 & 0.17 \end{array}\right]$ to $\left[\begin{array}{cc} 0.17 & 0.17\\ 0.17 & 0.17 \end{array}\right]$. In fact, the elements of the diagonal of **A** are constant and those of anti-diagonal linearly increase from 0 to 0.17, leading to more complex series. We moved a bivariate window—termed temporal window—with length 2000 samples and 20% overlap along this BAR(3) signal. The entropy of each bivariate temproal window is caculated. The results, depicted in Figure 5 show that when the time window is occupied at the beginning of the BAR(3) ($A=\left[\begin{array}{cc} 0.17 & 0\\ 0 & 0.17 \end{array}\right]$), the mvMDE_I_, mvMDE_II_, mvMDE_III_, and mvMDE values at higher scale factors are the smallest, showing the least complexity of BAR(3) in lower temporal windows, while their corresponding entropy values in the end of BAR(3) process ($A=\left[\begin{array}{cc} 0.17 & 0.17\\ 0.17 & 0.17 \end{array}\right]$) are the largest. It is worth noting that as described before, mvMDE_II_ needs a larger number of sample points to appropriately characterize the dynamics of signals. This fact can be observed in Figure 5, showing mvMDE_II_ is the least able to distinguish such changes.

### 4.2. Real Biomedical Datasets

**Figure 6:**
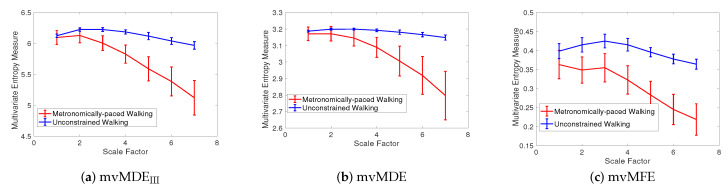
Mean value and SD of the results using (**a**) mvMDE_III_, (**b**) mvMDE, and (**c**) mvMFE for self-paced vs. metronomically-paced stride interval fluctuations.

**Figure 7:**
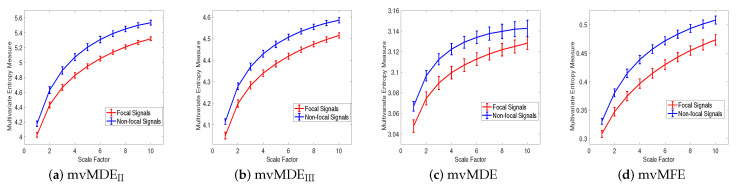
Mean value and SD of the results using (**a**) mvMDE_II_, (**b**) mvMDE_III_, (**c**) mvMDE, and (**d**) mvMFE for focal vs. non-focal time series.

**Figure 8:**
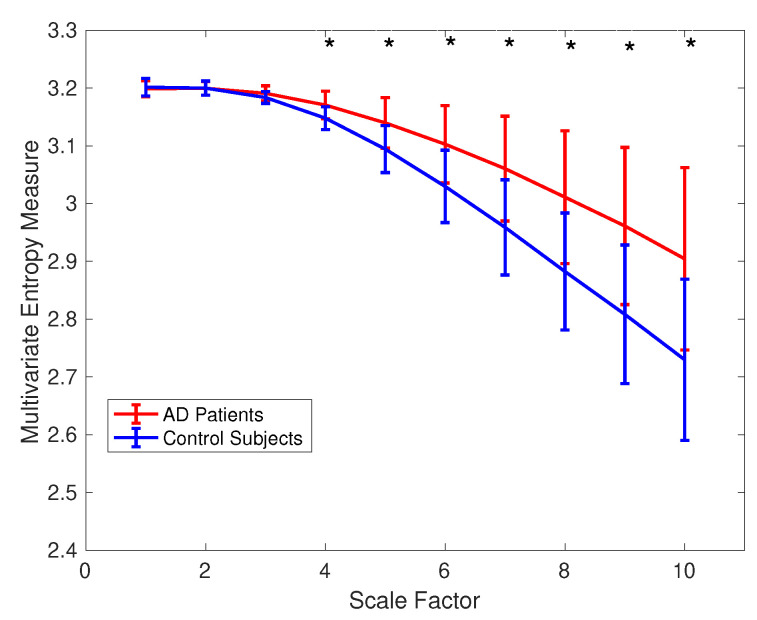
Mean value and SD of the results obtained by mvMDE computed from 36 AD patients versus 26 elderly controls for all the 148 channels. Red and blue respectively indicate AD patients and controls. The scales with *p*-values smaller than 0.001 are shown with *.

Discrimination of aged and diseased individuals’ from control or healthy subjects’ time series is a long-lasting challenge in the physiological complexity literature [8,10,17]. To this end, we use the mvMDE methods, in comparison with mvMFE as an improved version of mvMSE [17], to detect different types of dynamical variability of multivariate recordings of three physiological datasets. Of note is that we do not use the mvMDE_I_ for biomedical signals, because it does not take into account both the spatial and time domains at the same time.

(*1) Dataset of Stride Interval Fluctuations*: For the self-paced versus metronomically-paced stride interval fluctuations, the results obtained by the mvMDE_III_, mvMDE, and mvMFE, respectively depicted in Figure 6a–c, show that the self-paced unconstrained walk’s fluctuations have more complexity and greater long-range correlations than the metronomically-paced walk’s series, in agreement with those reportred in [2]. We did not use mvMDE_II_, as the signals do not follow the typical number of samples required for mvMDE_II_. To compare the results, the CV values for both the metronomically- and self-paced walk (MPW and SPW) at scale factor 4, as a trade-off between the long and short scales, are shown in Table 4. The CV values for the mvMDE_III_- and mvMDE-based profiles are smaller than those for mvMFE, showing the superiority of the proposed methods over mvMFE in terms of the stability of results. The smallest CV values are achieved by the mvMDE.

(*2) Dataset of Focal and Non-focal Brain Activity*: For the focal and non-focal EEG recordings, the results obtained by mvMDE_II_, mvMDE_III_, mvMDE, and mvMFE, respectively depicted in Figure 7a–d, show that the focal time series are less complex than the non-focal ones, in agreement with previous studies [40,43]. The CV values for the focal- and non-focal-based results at scale 6 are shown in Table 5. All the mvMDE-based CV values are smaller than those using mvMFE, showing more stability of the results obtained by the proposed methods. Moreover, the CV values for mvMDE are smaller than those for mvMDE_III_, and the latter ones are smaller than those for mvMDE_II_, suggesting that the mvMDE leads to more stable profiles.

(*3) Surface MEG Recordings in Alzheimer’s Disease*: To assess the ability of mvMDE, in comparison with mvMFE, we applied the methods to the 148-channel MEG signals to discriminate AD patients from controls. Because mvMFE needs to store a huge number of elements for a signal with a large number of channels, mvMFE was not able to simultaneously analyse all 148 time series. However, the results using mvMDE are depicted in Figure 8. It represents an advantage of mvMDE over mvMFE for signals with a large number of channels. To compare the mvMFE and mvMDE, we applied the methods to five main scalp regions, namely, anterior (17 channels), right (34 channels) and left lateral (34 channels), central (29 channels), and posterior (34 channels) areas, leading to the smaller number of channels to noticeably decrease the number of elements stored by the use of the mvMFE algorithm.

**Figure 9:**
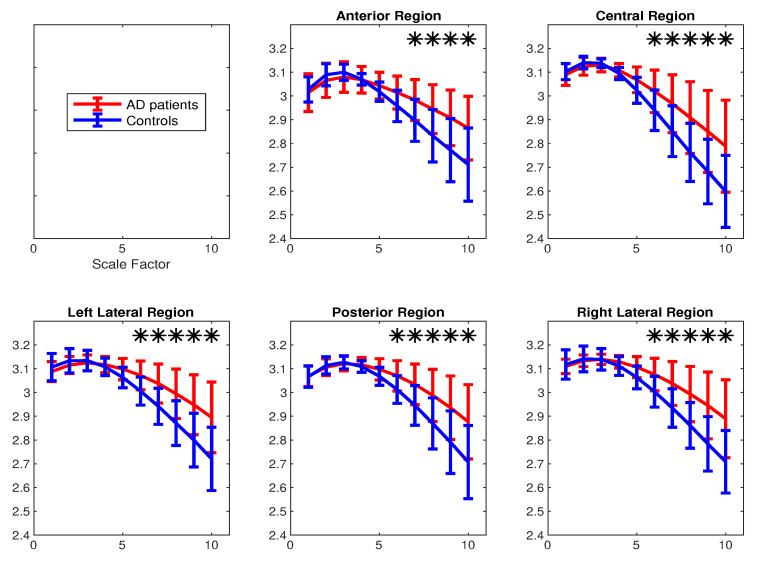

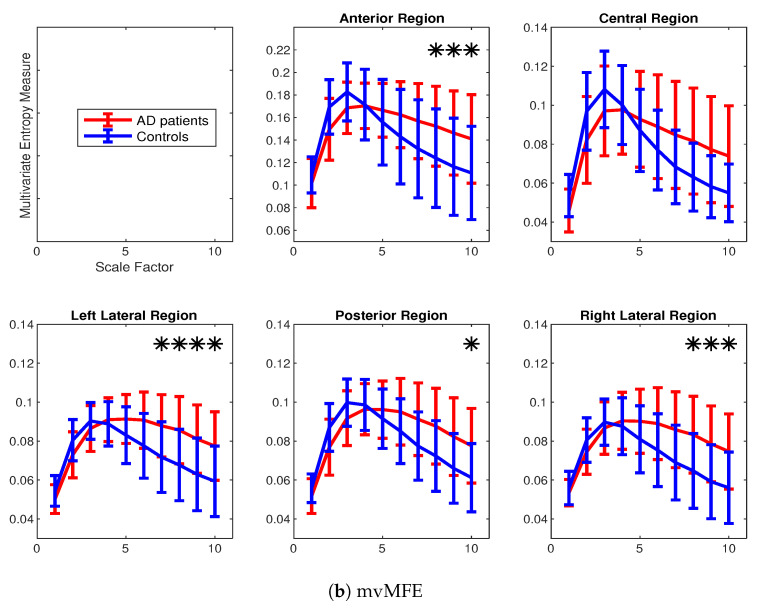
Mean value and SD of the results obtained by (**a**) mvMDE and (**b**) mvMFE computed from 36 AD patients versus 26 elderly age-matched controls over five scalp regions. Red and blue indicate AD patients and controls, respectively. The scale factors with *p*-values smaller than 0.001 are shown with *.

The average and SD of mvMDE and mvMFE values for five regions are respectively shown in Figure 9a,b. As can be seen in Figure 8 and Figure 9, the average mvMDE and mvMFE values for AD patients are smaller than those for controls at lower scale factors (short-time scale factors), while at higher scales, the AD subjects’ recordings have larger entropy values (long-time scale factors) for both the mvMFE and mvMDE, in agreement with [21,44,45]. Because the larger the number of channels, the smaller the mvMSE and similarly mvMFE values [21], the entropy values for anterior region are larger than those for the other four regions. It is worth noting that we only use mvMDE, as the signals do not follow the typical number of samples required for mvMDE_II_ and mvMDE_III_.

The Mann–Whitney *U*-test was used to assess the differences between the mvMDE and mvMFE profiles at each temporal scale for AD patients versus controls, because the mvMDE and mvMFE values at each scale factor did not follow a normal distribution. The temporal scales with *p*-values smaller than 0.001 are shown with * in Figure 8 and Figure 9. The *p*-values show that the mvMDE, compared with the mvMFE, significantly discriminated the controls from subjects with AD at a larger number of scale factors. Moreover, the smallest *p*-value was achieved by the mvMDE, evidencing the superiority of mvMDE over mvMFE.

The Hedges’ *g* effect size [46] was also used to quantify the differences between the entropy values for the AD patients’ vs. healthy controls’ MEGs for the five main brain regions [47]. The Hedges’ *g* test shows the difference between the means of two groups, divided by the weighted average of standard deviations for these two groups. The differences, illustrated in Table 6, show that the highest effect size is obtained by mvMDE, showing the advantage of this method over mvMFE.

On the whole, the profiles for the real datasets show the advantage of mvMDE_II_, mvMDE_III_, and mvMDE over mvMFE to discriminate different types of dynamics of multi-channel signals as well as the superiority of mvMDE over mvMFE in terms of ability to discriminate various dynamics of time series, computational time, and memory cost. As mentioned before, mvMPE does not consider the spatial domain. We have also refined the mvMPE [19] on the basis of mvMDE_II_, mvMDE_III_, and mvMDE. These approaches have the following advantages over the first version of mvMPE [19]: (1) they take into account both the spatial and time domains; (2) their results were more stable than the mvMPE-based ones; and (3) better distinguished different dynamics of multivariate signals. However, since the mvMDE methods are considerably faster, result in more stable profiles, and lead to larger differences between physiological conditions of recordings, for simplicity, we did not report the mvMPE-based results.

In this article, we proposed four implementations of the mvDE methods combined with the most commonly used coarse-graining process [3,8,17]. The key contribution of this study was introducing the mvDE methods. The alternative coarse-graining processes based on multivariate empirical mode decomposition [2,28,48,49,50], and FIR filters [28,51], though out of the scope of this paper, can be employed instead of the classical implementation of coarse-graining process used herein.

Our future study will aim at proposing the refined composite mvMDE (RCmvMDE) approaches according to [17]. Moreover, we will explore the mvMDE and RCmvMDE on other physiological and non-physiological time series. The similarity of two multi-channel signals based on mvMDE and cross-entropy [11] can also be developed as future work. An important step in making mvMDE a useful and stable metric is the mapping of the data to discrete set of integers via the normal cumulative distribution. Other mapping functions are available in [30]. The mvMDE method and its univariate form can also be generalized based on Renyi entropy [52].

## 5. Conclusions

To quantify the complexity of multivariate time series, we built four diverse alternative implementations of mvMDE as further developments of our recently introduced MDE [27]. These insights help towards a comprehensive understanding of four strategies to extend a univariate-based entropy method to its multivariate versions and therefore, provide invaluable information for future studies on multivariate time series. Although mvMDE was the best algorithm in terms of ability to discriminate dynamics of multivariate signals, computational time, and memory cost, the simpler alternatives (mvDE_I_ to mvDE_III_) may still be useful in some settings.

We assessed their performance on the correlated and uncorrelated multivariate noise signals, the bivariate AR time series, and three physiological datasets. The results showed the similar behavior of mvMSE-, mvMFE-, and mvMDE-based profiles. However, mvMDE had the following advantages over the existing methods: (1) it was faster than the existing methods; (2) mvMDE, in comparison with mvMSE and mvMFE, resulted in more stable profiles; (3) mvMDE better discriminated different kinds of biomedical signals; (4) for short multivariate time series (300 sample points), mvMDE did not result in undefined values; and (5) mvMDE, compared with mvMSE and mvMFE, needed to store a considerably smaller number of elements.

Overall, we expect the mvMDE approach to play a key role in the assessment of complexity in multivariate time series.

## Figures and Tables

**Table 1:** Ability to deal with spatial domain and characterization of short signals (300 sample points), typical number of elements to be stored, and typical number of samples needed for each of the mvSE, mvFE, and mvDE algorithms for a *p*-channel signal with length *N* sample points.

Methods	Spatial Domain	Short Signals	Typical Number of Elements Stored	Typical Number of Samples
mvSE [3]	yes	undefined	Np2+Np(pm+1)	$10^{m}<N$
mvFE [17]	yes	unreliable	Np2+Np(pm+1)	$10^{m}<N$
mvPE [19] and mvWPE [20]	no	reliable	m!+Np	m!<N
mvDE_I_	no	reliable	$c^{m}+Np$	$\frac{c^{m}}{p}<N$
mvDE_II_	yes	unreliable	$c^{mp}+Np$	$c^{mp}<N$
mvDE_III_	yes	unreliable	$c^{m+p-1}+Np$	$\frac{c^{m+p-1}}{p}<N$
mvDE	yes	reliable	$c^{m}+Np$	cmmpm<N

**Table 2:** CV values of the proposed and existing multivariate multiscale entropy-based analyses at scale factor 10 for the uncorrelated trivariate 1/f noise and WGN.

Time Series	mvMDE_I_	mvMDE_II_	mvMDE_III_	mvMDE	mvMSE	mvMFE
All three channels contain 1/f noise	0.0028	0.0025	0.0037	0.0022	0.0405	0.0355
Two channels contain 1/f noise and one contains WGN	0.0042	0.0032	0.0036	0.0044	0.0283	0.0274
One channel contains 1/f noise and two contain WGN	0.0066	0.0052	0.0058	0.0061	0.0305	0.0292
All three channels contain WGN	0.0072	0.0080	0.0092	0.0101	0.0232	0.0211

**Table 3:** Computational time of the mvMSE, mvMFE, and mvMDE algorithms with $\tau_{max}=10$.

Number of Channels and Samples	mvMSE	mvMFE	mvMDE_I_	mvMDE_II_	mvMDE_III_	mvMDE
2 channels and 1000 samples	0.051 s	0.066 s	0.014 s	0.023 s	0.026 s	0.020 s
2 channels and 3000 samples	0.237 s	0.296 s	0.035 s	0.057 s	0.068 s	0.052 s
2 channels and 10,000 samples	1.821 s	2.016 s	0.111 s	0.190 s	0.223 s	0.181 s
5 channels and 1000 samples	0.209 s	0.223 s	0.028 s	43.096 s	0.490 s	0.050 s
5 channels and 3000 samples	1.129 s	1.204 s	0.080 s	82.246 s	1.137 s	0.137 s
5 channels and 10,000 samples	9.432 s	9.801 s	0.260 s	218.553 s	3.343 s	0.491 s
8 channels and 1000 samples	0.489 s	0.501 s	0.042 s	out of memory error	65.560 s	0.086 s
8 channels and 3000 samples	2.973 s	2.906 s	0.124 s	out of memory error	150.122 s	0.243 s
8 channels and 10,000 samples	27.993 s	25.951 s	0.398 s	out of memory error	363.752 s	0.824 s

**Table 4:** CV values of the entropy results at scale factor 4 using mvMDE_III_, mvMDE, and mvMFE for self-paced walk (SPW) vs. metronomically-paced walk (MPW).

Stride Interval Fluctuations	mvMFE	mvMDE_III_	mvMDE
Self-paced walk	0.040	0.005	0.002
Metronomically-paced walk	0.116	0.025	0.019

**Table 5:** CV values of the entropy results at scale factor 6 using mvMDE_II_, mvMDE_III_, mvMDE, mvMSE, and mvMFE for focal vs. non-focal EEG recordings.

Signals	mvMSE	mvMFE	mvMDE_II_	mvMDE_III_	mvMDE
focal EEGs	0.019	0.019	0.006	0.003	0.002
Non-focal EEGs	0.021	0.015	0.008	0.003	0.002

**Table 6:** Differences between results for AD patients’ vs. healthy controls’ MEGs obtained by mvMFE and mvMDE for five main brain regions based on the Hedges’ *g* effect size.

Region-Method	Scale 1	Scale 2	Scale 3	Scale 4	Scale 5	Scale 6	Scale 7	Scale 8	Scale 9	Scale 10
Anterior-mvMFE	0.36	0.73	0.57	0.04	0.33	0.53	0.63	0.70	0.72	0.73
Central-mvMFE	0.68	0.67	0.49	0.10	0.23	0.48	0.65	0.76	0.79	0.83
Left lateral-mvMFE	0.53	0.64	0.34	0.18	0.60	0.83	0.92	0.98	0.97	0.98
Posterior-mvMFE	0.46	0.72	0.58	0.16	0.30	0.57	0.73	0.78	0.82	0.85
Right lateral-mvMFE	0.30	0.50	0.22	0.18	0.53	0.71	0.84	0.92	0.97	0.95
Anterior-mvMDE	0.18	0.37	0.36	0.03	0.49	0.80	0.95	1.02	1.06	1.04
Central-mvMDE	0.29	0.45	0.29	0.48	0.78	0.88	0.97	1.01	1.03	1.04
Left lateral-mvMDE	0.37	0.40	0.24	0.24	0.77	1.07	1.17	1.20	1.19	1.19
Posterior-mvMDE	0.05	0.19	0.18	0.24	0.67	0.90	1.015	1.05	1.06	1.06
Right lateral-mvMDE	0.15	0.19	0.00	0.51	0.90	1.05	1.14	1.18	1.20	1.16
